# Estimates of 30-day postoperative pulmonary complications after gastrointestinal endoscopic procedures: A retrospective cohort analysis of a health system population

**DOI:** 10.1371/journal.pone.0299137

**Published:** 2024-02-23

**Authors:** Zyad J. Carr, Judy Li, Daniel Agarkov, Makenzie Gazura, Kunal Karamchandani

**Affiliations:** 1 Department of Anesthesiology, Yale University School of Medicine, New Haven, Connecticut, United States of America; 2 Yale University School of Medicine, New Haven, Connecticut, United States of America; 3 Frank H. Netter MD School of Medicine, Quinnipiac University, North Haven, Connecticut, United States of America; 4 Department of Anesthesiology and Pain Management, University of Texas Southwestern Medical Center, Dallas, Texas, United States of America; University of Leeds, UNITED KINGDOM

## Abstract

The incidence of 30-day postoperative pulmonary complications (PPC) of gastrointestinal endoscopic procedures (GIEP) are not well characterized in the literature. The primary aim of this study was to identify the incidence of 30-day PPC after GIEP within a large healthcare system. We conducted a retrospective cohort study of 5377 patients presenting for GIEP between January 2013 and January 2022. Our primary outcome was the Agency for Healthcare Research and Quality PPC composite (AHRQ-PPC). Secondary outcomes were sub-composites derived from the AHRQ-PPC; including pneumonia (AHRQ-PNA), respiratory failure (AHRQ-RF), aspiration pneumonia/ pneumonitis (AHRQ-ASP) and pulmonary emboli (AHRQ-PE). We performed propensity score matching (PSM) followed by multivariable logistic regression to analyze primary and secondary outcomes. Inpatients had higher 30-day AHRQ-PPC (6.0 vs. 1.2%, p<0.001), as well as sub-composite AHRQ-PNA (3.2 vs. 0.7%, p<0.001), AHRQ-RF (2.4 vs. 0.5%, p<0.001), and AHRQ-ASP (1.9 vs. 0.4%, p<0.001). After PSM adjustment, pre-procedural comorbidities of electrolyte disorder [57.9 vs. 31.1%, ORadj: 2.26, 95%CI (1.48, 3.45), p<0.001], alcohol abuse disorder [16.7 vs. 6.8%, OR_adj_: 2.66 95%CI (1.29, 5.49), p = 0.01], congestive heart failure (CHF) [22.3 vs. 8.7%, OR_adj_: 2.2 95%CI (1.17, 4.15), p = 0.02] and pulmonary circulatory disorders [21 vs. 16.9%, OR_adj_: 2.95, 95%CI (1.36, 6.39), p = 0.01] were associated with 30-day AHRQ-PPC. After covariate adjustment, AHRQ-PPC was associated with upper endoscopy more than lower endoscopy [5.9 vs. 1.0%, OR_adj_: 3.76, 95%CI (1.85, 7.66), p<0.001]. When compared to gastroenterologist-guided conscious sedation, anesthesia care team presence was protective against AHRQ-PPC [3.7 vs. 8.4%, OR_adj_: 0.032, 95%CI (0.01, 0.22), p<0.001] and AHRQ-ASP [1.0 vs. 3.37%, OR_adj_: 0.002, 95%CI (0.00, 0.55), p<0.001]. In conclusion, we report estimates of 30-day PPC after GIEP across inpatient and outpatient settings. Upper endoscopic procedures confer a higher risk, while the presence of an anesthesia care team may be protective against 30-day PPC.

## Introduction

The incidence of postoperative pulmonary complications (PPC) occurring within 30 days after gastrointestinal endoscopic procedures (GIEP) is not well characterized in the literature. Preprocedural risk estimation and assessment for GIEP are critical to reducing peri-procedural complications [1]. A prospective observational study, utilizing questionnaires after GIEP with endoscopist-driven sedation, observed a significant incidence of clinically evident respiratory infection [2]. Although dependent on individual reported variables, combined PPC in all surgical populations may range between 1–10.9% [3–5]. With increasing efforts at decreasing hospital length of stay after procedure, postoperative infectious complications have been increasingly observed to occur after discharge and increase the risk of readmission, thus characterizing 30-day outcomes may better estimate true incidence of perioperative complications [6]. Agency for Healthcare Research and Quality (AHRQ) composite patient safety indicators (PSI) have shown moderate sensitivity and high specificity for postoperative procedural complications [7]. Our primary aim was to provide risk estimates for 30-day PPC after GIEP in a diverse health system population. To this end, we utilized ICD-10 coding for the AHRQ postoperative pulmonary complications (AHRQ-PPC) [8]. The AHRQ-PPC is a broad composite of International Classification of Disease (ICD-10) and current procedural terminology^®^ (CPT) codes for respiratory failure, infectious pneumonia, pneumonitis, pulmonary embolus, pneumothorax, and pulmonary aspiration. For exploratory secondary outcomes, we created focused sub-composites derived from the AHRQ-PPC that provide diagnostic clusters for respiratory failure (AHRQ-RF), infectious pneumonia (AHRQ-PNA), pulmonary aspiration pneumonitis/pneumonia (AHRQ-ASP), and pulmonary embolus (AHRQ-PE). We analyzed pre-admission risk factors for AHRQ-PPC, the role of anesthesia care team presence during GIEP, risk of upper vs. lower endoscopy and compared outpatient vs. inpatient endoscopy.

## Materials and methods

### Study population

**Fig 1:**
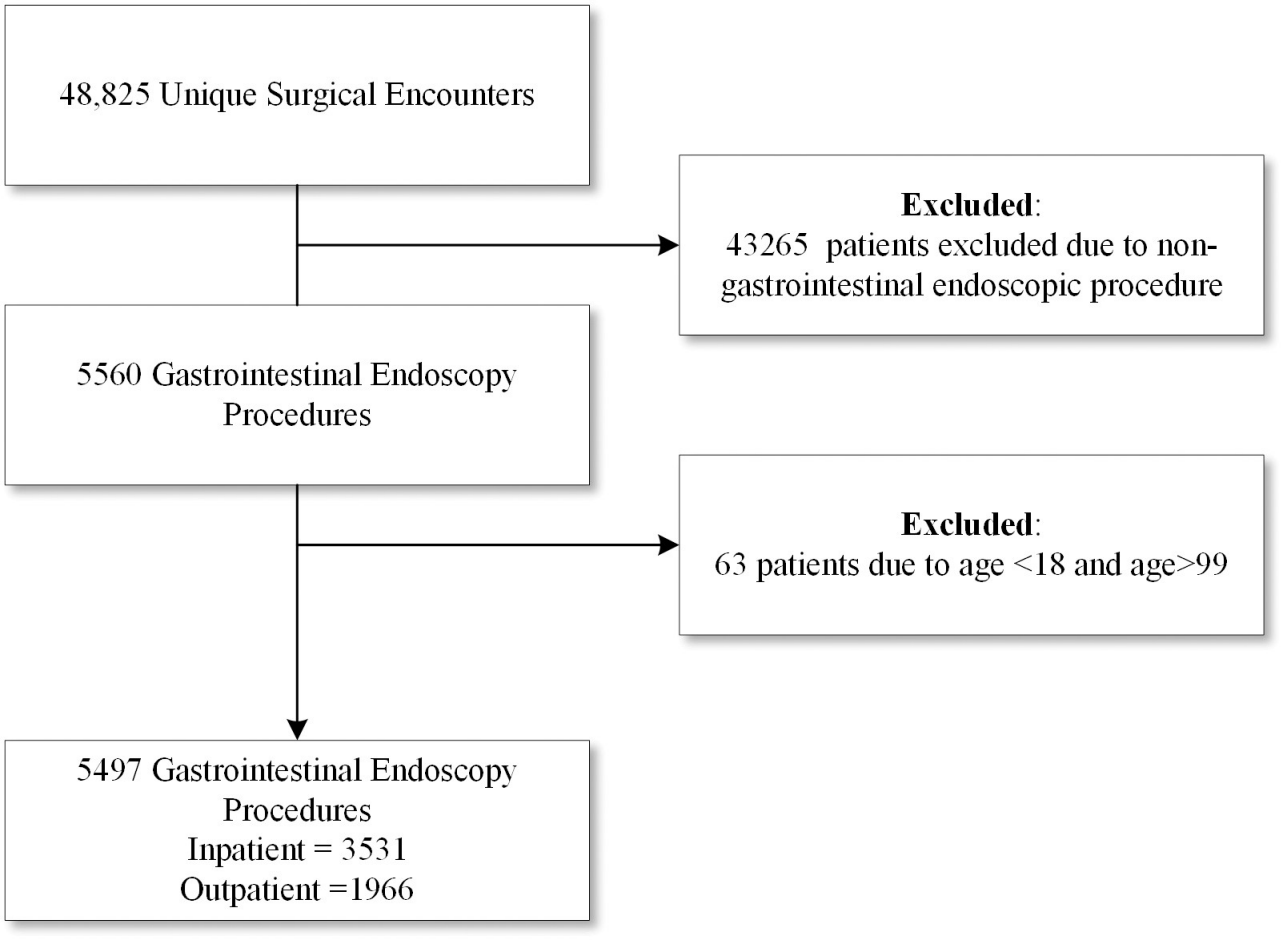
CONSORT flow diagram of patient eligibility.

We conducted a retrospective cohort study of patients who underwent GIEP between January 1, 2013, and January 1, 2022, within the Yale-New Haven Hospital system which includes a non-profit, 1,541 bed tertiary medical center, three community hospitals and three endoscopy centers located in Connecticut, USA. The study was approved by the Yale School of Medicine Institutional Review Board (Study #2000032725) with a waiver of informed consent. Authors had access to data of individual participants after data collection. This manuscript follows the recommendations published by the Strengthening the Reporting of Observational Studies in Epidemiology (STROBE) Statement guidelines for observational studies in epidemiology (Fig 1) [9].

GIEP was defined as upper or lower endoscopic procedures performed by the gastrointestinal service with the involvement of the anesthesia care team (comprised of a supervising anesthesiologist with resident or certified nurse anesthetist) or gastroenterologist guided conscious sedation (comprised of a gastroenterologist and nursing team). We extracted age, gender, body mass index (BMI), American Society of Anesthesiologists physical status (ASA-PS I-IV), endoscopic procedure type (upper vs. lower endoscopy), procedural location (inpatient vs. outpatient setting), Elixhauser composite comorbidities, CPT and ICD-10 codes for primary and secondary outcomes. We then performed manual chart review of the 233 unique patients with 30-day PPC to determine if the complication occurred during hospital admission or in the 30 days following discharge.

### Study outcomes

The primary outcome was AHRQ-PPC, and the secondary outcomes were sub-composites (AHRQ-RF, AHRQ-PNA, AHRQ-ASP and AHRQ-PE). Individual ICD-10 or CPT components of the AHRQ-PPC composite and sub-composites are described in S1 Table. We analyzed the sub-groupings of anesthesia care team vs. gastroenterologist-guided conscious sedation to the primary outcome and inpatient vs. outpatient GIEP to analyze for differences in AHRQ-PPC and sub-composite incidences.

### Statistical analysis

**Fig 2:**
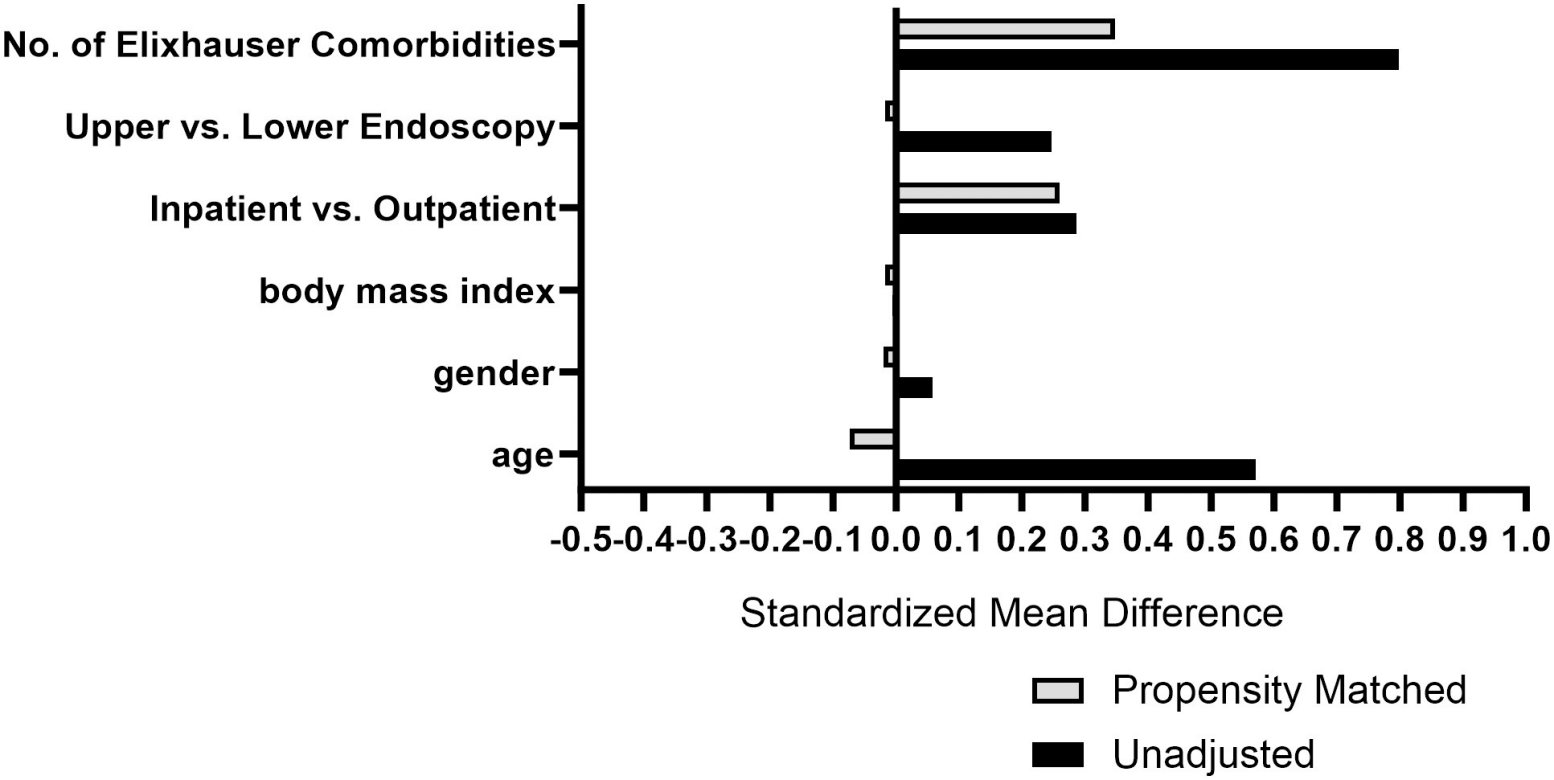
Standardized mean differences in the study cohort before and after propensity score adjustment.

To provide generalized risk estimates, descriptive statistics were generated with categorical variables provided as absolute frequencies (number/percent) and continuous variables as averages (mean/SD). Univariate analysis was performed and subsequently compared using Chi-square or two-sample t-test as indicated. To enhance adjustment for primary outcome assessment and due to the high number of missing values for ASA physical status (ASA-PS), a measure of disease severity prior to surgical procedure, we performed and examined a receiver operating characteristic (ROC) curve for total number of Elixhauser comorbidities and its relationship with 30-day mortality. We observed an areas under the ROC curve (AUC) of 0.966 (number of Elixhauser comorbidities) and 0.920 (categorized number of Elixhauser comorbidities), demonstrating that increasing number of Elixhauser comorbidities are a useful predictor of overall disease severity and 30-day perioperative mortality. To balance clinically relevant baseline covariates, we generated propensity scores using nearest-neighbor matching without replacement and a caliper width of 0.10. We entered age, gender, BMI, categorized number of Elixhauser comorbidities (0–5, 6–10, >10), inpatient vs. outpatient status, and upper vs. lower endoscopy (Fig 2). For the exploratory analysis of pre-procedural risk predictors for AHRQ-PPC, we performed propensity score matching (PSM) to balance between-subject selection bias in patients with and without AHRQ-PPC. The performance of PSM reduces the risk of selection bias by providing optimal assessment of treatment effect via balancing of clinically relevant baseline covariates [10]. The propensity matched cohorts were then analyzed using multivariate logistic regression to compare pre-procedural risk predictors’ association with 30-day AHRQ-PPC. Hosmer and Lemeshow Test demonstrated acceptable model fit (p = 0.191). For sub-group analysis, we performed multivariable logistic regression modeling adjusted for age, gender, BMI, number of comorbidities, length of procedure, upper vs. lower endoscopy and inpatient vs. outpatient location. Acceptable model fit was observed (Nagelkerke R2 = 0.54). For the purposes of the exploratory analysis, we accepted a p value of <0.05 for significance. Given the exploratory nature of the analysis, adjustment for multiple outcomes was not performed. SPSS 28.0 (SPSS Inc., IL, USA) was used for analysis.

## Results

### Incidence of 30-day PPC in the overall population

Baseline characteristics of the study population are provided in Table 1. The overall incidence of 30-day AHRQ-PPC in the study population was 4.2%. When compared to patients that did not develop PPC, those that did were older (66.8 vs. 58.23 years old, p<0.001), had higher proportion of patients with ASA-IV physical status (18.90 vs. 3.1%, p<0.001), higher number of Elixhauser comorbidities (5.1 vs. 1.29, p<0.001) but had similar gender demographics (52.4 vs. 58.2, % female, p = 0.77), BMI (30.01 vs. 30.1 kg/m^2^, p = 0.12) and ethnic backgrounds (p = 0.21). Patients who developed the primary outcome had higher incidence of upper endoscopies (92.7% (217/233), p<0.001), 90.5% were associated with endoscopies performed in an inpatient setting, and 26.6% of AHRQ-PPC were recorded after discharge. Both number and individual pre-admission Elixhauser comorbidities were higher for those that developed PPC when compared to patients that did not, except for pre-procedural comorbidities of asthma, peptic ulcer disease, liver disease, end-stage renal disease, uncomplicated diabetes mellitus, rheumatological disease and nicotine use disorder.

**Table 1:** Baseline characteristics and univariate analysis of the study population.

Variable	No AHRQ-PPC (N = 5144)		AHRQ-PPC (N = 233)		Total (N = 5377)		P-value*
**Age** in years, mean (SD)	58.2	±16.1	66.8	±16.7	58.6	±16.1	**<0.001**
**Gender** n (%)							0.077
Female	2994	58.2%	122	52.4%	3115	58.0%	
Male	2150	41.8%	111	47.6%	2261	42.0%	
**Weight**							
BMI (kg/meter^2^) mean (SD)	30.1	±8.0	29.2	±7.7	30.01	±8.1	0.12
**Ethnicity**							0.21
White or Caucasian**	3675	71.4%	184	79.0%	3859	71.8%	
Black or African American	1005	19.5%	42	18.0%	1047	19.5%	
Other/Not Listed	253	4.9%	4	1.7%	257	4.8%	
Asian	62	1.2%	2	0.9%	64	1.2%	
Other	58	1.1%	1	0.4%	59	1.1%	
Patient Refused	60	1.2%	0	0.0%	60	1.1%	
American Indian or Alaska Native	13	0.3%	0	0.0%	13	0.2%	
Unknown	9	0.2%	0	0.0%	9	0.2%	
Native Hawaiian or Other Pacific Islander	5	0.1%	0	0.0%	5	0.1%	
Other Pacific Islander	4	0.1%	0	0.0%	4	0.1%	
**American Society of Anesthesiologists—PS**							**<0.001**
Healthy (ASA-I)	202	3.9%	2	0.9%	204	3.8%	
Mild Systemic Disease (ASA-II)	1509	29.3%	14	6.0%	1523	28.3%	
Severe Systemic Disease (ASA-III)	1740	33.8%	79	33.9%	1819	33.8%	
Incapacitating Disease (ASA-IV)	158	3.1%	44	18.9%	202	3.8%	
Not recorded	1535	29.8%	94	40.3%	1629	30.3%	
**Procedure Type**							**<0.001**
Upper Endoscopic Procedures	3451	67.1%	216	92.7%	3667	68.2%	
Lower Endoscopic Procedures	1693	32.9%	17	7.3%	1710	31.8%	
**Procedure Time**							
Total time, in minute: second, mean (SD)	48:04	±27:06	62:45	±37:50	48:32	±27:39	**<0.001**
**Anesthesia Type**							**<0.001**
General anesthesia with or without an airway	4275	85.9%	166	72.2%	4441	82.6%	
Nursing/Gastroenterology conscious sedation	702	14.1%	64	27.8%	766	14.2%	
Not recorded					170	3.2%	
**Procedure Location**							**<0.001**
Inpatient Setting	3267	63.5%	210	90.1%	3477	64.7%	
Outpatient Setting	1877	36.5%	23	9.9%	1900	35.3%	
**Selected Pre-Procedural Detailed Elixhauser Comorbidities**							
** Total Number of Comorbidities**	1.29	±1.81	5.1	±3.33	1.46	±2.05	**<0.001**
**Neurological**							
Cerebrovascular Disease	101	2.0%	42	18.0%	143	2.7%	**<0.001**
Transient Ischemic Attack	16	0.3%	4	1.7%	20	0.4%	**<0.001**
Stroke	31	0.6%	22	9.4%	53	1.0%	**<0.001**
**Cardiac**							
Hypertension, Uncomplicated	537	10.4%	52	22.3%	589	11.0%	**<0.001**
Hypertension, Complicated	122	2.4%	36	15.5%	158	2.9%	**<0.001**
Valve Disease							
Congestive Heart Failure	126	2.4%	52	22.3%	178	3.3%	**<0.001**
Myocardial Infarction	76	1.5%	23	9.9%	99	1.8%	**<0.001**
Pulmonary Circulatory Disease	29	0.6%	35	15.0%	64	1.2%	**<0.001**
Atrial Fibrillation	96	1.9%	20	8.6%	116	2.2%	**<0.001**
Lipid Disorder	668	13.0%	48	20.6%	716	13.3%	**<0.001**
Peripheral Vascular Disease	96	1.9%	28	12.0%	124	2.3%	**<0.001**
**Pulmonary**							
Chronic Obstructive Pulmonary Disease	112	2.2%	21	9.0%	133	2.5%	**<0.001**
Asthma	226	4.4%	15	6.4%	241	4.5%	0.14
**Gastrointestinal**							
Peptic Ulcer Disease	343	6.7%	28	12.0%	371	6.9%	0.002
Liver disease	404	7.5%	2	20.0%	406	7.6%	0.13
**Renal**							
Chronic Kidney Disease	123	2.4%	30	12.9%	153	2.8%	**<0.001**
End Stage Renal Disease	8	0.2%	2	0.9%	10	0.2%	0.015
Electrolyte Disorder	374	7.3%	135	57.9%	509	9.5%	**<0.001**
**Endocrine**							
Diabetes Mellitus, uncomplicated	240	4.7%	15	6.4%	255	4.7%	0.213
Diabetes Mellitus, complicated	110	2.1%	19	8.2%	129	2.4%	**<0.001**
Hypothyroidism	163	3.2%	25	10.7%	188	3.5%	**<0.001**
**Hematological**							
Blood Loss Anemia	114	2.2%	26	11.2%	140	2.6%	**<0.001**
Iron Deficiency Anemia	251	4.9%	27	11.6%	278	5.2%	**<0.001**
Coagulopathy	103	2.0%	35	15.0%	138	2.6%	**<0.001**
**Rheumatological**							
Rheumatological Disorder	35	0.7%	4	1.7%	39	0.7%	0.068
**Oncological**							
Solid Tumor	229	4.5%	24	10.3%	253	4.7%	**<0.001**
Metastatic Disease	81	1.6%	14	6.0%	95	1.8%	**<0.001**
Lymphoma	17	0.3%	4	1.7%	21	0.4%	**<0.001**
Weight Loss	237	4.6%	51	21.9%	288	5.4%	**<0.001**
**Social History**							
Nicotine Disorder	242	4.7%	19	8.2%	261	4.9%	0.017
Alcohol Disorder	134	2.6%	39	16.7%	173	3.2%	**<0.001**
Illicit Drug Use	100	1.9%	19	8.2%	119	2.2%	**<0.001**
Obesity	505	9.8%	40	17.2%	545	10.1%	**<0.001**

Abbreviations: ASA-PS; American Society of Anesthesiologists Physical Status; BMI; body mass index

*Chi-square, t-test as appropriate, two-tailed p value. Based on multiple outcomes, a suggested Bonferroni adjusted p-value for significance is p<0.0012.

**Includes 9.8% reporting Hispanic ethnicity

### Differences between inpatient vs. outpatient study cohorts

Unadjusted differences between inpatient and outpatient cohorts are described in Table 2. Age (58.78 vs. 58.17 years old, p = 0.191) and gender (58.7 vs. 56.7% female, p = 0.146) were similar, but inpatients had higher BMI (30.31 vs. 29.52 m/kg^2^, p<0.001). Inpatients had a higher incidence of AHRQ-PPC (6.00 vs. 1.20%, p<0.001). Significant differences were observed in AHRQ-PNA (3.20 vs. 0.70%, p<0.001), AHRQ-RF (2.40 vs. 0.50%, p<0.001), AHRQ-ASP (1.90 vs. 0.40%, p<0.001), but not AHRQ-PE (0.60 vs. 0.40%, p = 0.01). Lastly, inpatients had higher rates of readmission within 30 days (17.41 vs. 2.70%, p<0.001) and higher 30-day mortality (1.00 vs. 0.20%, p<0.001).

**Table 2:** Comparison of peri-procedural outcomes in inpatient vs. outpatient study cohorts.

Variable	Inpatient* (N = 3476)		Outpatient** (N = 1900)		Total (N = 5377)		P-value†
**Baseline Demographics**							
Age, in years, mean, SD‡	58.9	±17.4	58.1	±13.6	58.6	±16.2	0.133
**Weight**							
BMI, kg/meter^2^, mean (SD)	30.3	±8.4	29.5	±7.2	30.1	±8.1	**0.005**
**Gender**							0.164
Female	2039	58.6%	1077	56.7%	3116	58.0%	
Male	1438	41.4%	823	43.3%	2261	42.0%	
**Number of Elixhauser Comorbidities**							
Comorbidities, mean SD	1.8	±2.3	0.8	±1.4	1.5	±2.1	**<0.001**
**30-Day Peri-procedural Pulmonary Complications**							
AHRQ-PPC	210	6.0%	23	1.2%	233	4.3%	**<0.001**
AHRQ-PNA	110	3.2%	14	0.7%	124	2.3%	**<0.001**
AHRQ-RF	82	2.4%	9	0.5%	91	1.7%	**<0.001**
AHRQ-ASP	65	1.9%	8	0.4%	73	1.4%	**<0.001**
AHRQ-PE	20	0.6%	2	0.1%	22	0.4%	0.01
**30-Day Major Complications**							
Readmission w/in 30 days	601	17.3%	29	1.5%	630	11.7%	**<0.001**
Mortality w/in 30 days	37	1.0%	3	0.2%	40	0.7%	**<0.001**

*aggregated quaternary care facility and ancillary community hospitals

**stand-alone endoscopy centers.

^†^Chi-Square or t-test as appropriate, p-value denotes two-tailed test.

^‡^number percentage (%) unless otherwise noted.

Abbreviations: AHRQ: Agency for Healthcare Research & Quality; PPC: postoperative pulmonary complications; PNA: pneumonia; RF: respiratory failure; ASP: aspiration; DVT-PE: deep vein thrombosis/pulmonary embolism; BMI; body mass index

### Pre-admission co-morbidities associated with AHRQ-PPC

Table 3 provides the PSM analysis of the study cohort and Fig 2 describes standardized mean differences before and after PSM adjustment. After adjustment, pre-procedural comorbidities of electrolyte disorder [57.9 vs. 31.1%, ORadj: 2.26, 95%CI (1.48, 3.45), p<0.001], alcohol abuse disorder [16.7 vs. 6.8%, OR_adj_: 2.66 95%CI (1.29, 5.49), p = 0.01], CHF [22.3 vs. 8.7%, OR_adj_: 2.2 95%CI (1.17, 4.15), p = 0.02] and pulmonary circulatory disorders [21 vs. 16.9%, OR_adj_: 2.95, 95%CI (1.36, 6.39), p = 0.01] were associated with 30-day AHRQ-PPC.

**Table 3:** Univariate and multivariable analysis of 30-day postoperative pulmonary complications pre-procedural risk factors of the propensity score matched cohort.

Variable	No PPC* (n = 219)		PPC (n = 233)		p value	Risk Ratio	Lower CI	Upper CI
	Number	%	Number	%				
**Cardiovascular**								
Cardiac Valve Disease	21	9.6	53	22.7	**<0.001**	1.85	1.27	2.68
Pulmonary Circulatory Disorder	10	4.6	35	15.0	**<0.001**	2.31	1.33	4.02
Arrhythmia	44	20.1	61	26.2	0.13	1.20	0.94	1.54
Atrial Fibrillation	24	11.0	20	8.6	0.40	0.88	0.66	1.17
Congestive Heart Failure	19	8.7	52	22.3	**<0.001**	1.96	1.32	2.92
Uncomplicated HTN	54	24.7	52	22.3	0.56	0.94	0.75	1.16
Complicated HTN	29	13.2	36	15.5	0.50	1.10	0.82	1.47
Myocardial Infarction	12	5.5	23	9.9	0.08	1.45	0.91	2.31
Arrhythmia	44	20.1	61	26.2	0.13	1.20	0.94	1.54
Atrial Fibrillation	24	11.0	20	8.6	0.40	0.88	0.66	1.17
Bradyarrhythmia	3	1.4	4	1.7	0.77	1.13	0.48	2.68
Lipid Disorder	47	21.5	48	20.6	0.82	0.97	0.77	1.23
Peripheral Vascular Disease	23	10.5	28	12.0	0.61	1.08	0.79	1.49
**Pulmonary**								
Pulmonary Disease	37	16.9	49	21.0	0.26	1.16	0.89	1.51
Chronic Obstructive Pulmonary Disease	22	10.0	21	9.0	0.71	0.94	0.69	1.28
Asthma	15	6.8	15	6.4	0.86	0.97	0.67	1.40
**Neurological**								
Neurological Disease	28	12.8	44	18.9	0.08	1.29	0.95	1.76
Cerebrovascular Disease	22	10.0	42	18.0	**0.02**	1.48	1.04	2.10
Transient Ischemic Attack	3	1.4	4	1.7	0.76	1.13	0.48	2.68
Intracranial Hemorrhage	6	2.7	13	5.6	0.13	1.56	0.80	3.04
Stroke	10	4.6	22	9.4	**0.04**	1.59	0.94	2.69
Paralysis	6	2.7	10	4.3	0.37	1.30	0.69	2.47
**Gastrointestinal**								
Liver Disease	41	18.7	59	25.3	**0.09**	1.23	0.95	1.59
Peptic Ulcer Disease	26	11.9	28	12.0	0.96	1.01	0.75	1.35
**Renal**								
Electrolyte Disorder	68	31.1	135	57.9	**<0.001**	1.81	1.46	2.25
Renal Failure	30	13.7	34	14.6	0.79	1.04	0.79	1.38
End-stage Renal Disease	1	0.5	2	0.9	0.60	1.46	0.29	7.24
**Endocrine**								
Hypothyroidism	17	7.8	25	10.7	0.28	1.22	0.83	1.78
Uncomplicated Diabetes Mellitus	27	12.3	15	6.4	**0.03**	0.73	0.57	0.93
Complicated Diabetes Mellitus	20	9.1	19	8.2	0.71	0.94	0.68	1.30
**Psychiatric**								
Psychosis	3	1.4	12	5.2	**0.03**	2.47	0.89	6.83
Depression	25	11.4	37	15.9	0.17	1.23	0.90	1.70
**Hematological-Oncological**								
Solid Tumor	42	19.2	24	10.3	**0.01**	0.82	0.58	0.89
Metastatic Disease	16	7.3	14	6.0	0.58	0.90	0.64	1.28
Lymphoma	1	0.5	4	1.7	0.20	2.44	0.42	14.11
Acute blood loss anemia	18	8.2	26	11.2	0.29	1.20	0.83	1.12
Iron Deficiency anemia	22	10.0	27	11.6	0.60	1.09	0.79	1.51
Coagulopathy	19	8.7	35	15.0	**0.04**	1.43	0.98	2.08
Rheumatoid Disease	5	2.3	4	1.7	0.67	0.87	0.48	1.57
**Infectious Disease**								
Human Immunodeficiency Virus	0	0.0	0	0.0	n/a	n/a	n/a	n/a
**Social History**								
Weight Loss	37	16.9	51	21.9	0.18	1.19	0.91	1.55
Alcohol Abuse Disorder	15	6.8	39	16.7	**0.00**	1.85	1.19	2.87
Obesity	28	12.8	40	17.2	0.19	1.21	0.89	1.63
Illicit Drug Use Disorder	15	6.8	19	8.2	0.60	1.11	0.75	1.64
Nicotine Use Disorder	23	10.5	19	8.2	0.39	0.87	0.65	1.17
**Multivariable Logistic Regression Analysis of Pre-Procedural Risk Predictors**								
	No PPC (n = 219)		PPC (n = 233)		p value	Odds Ratio	Lower CI	Upper CI
	Number	%	Number	%				
Alcohol Abuse Disorder	15	6.8	39	16.7	**0.01**	2.66	1.29	5.49
Electrolyte Disorders	68	31.1	135	57.9	**<0.001**	2.26	1.48	3.45
Congestive Heart Failure	19	8.7	52	22.3	**0.02**	2.20	1.17	4.15
Pulmonary Circulatory Disorder	37	16.9	49	21.0	**0.01**	2.95	1.36	6.39

*denotes AHRQ-PPC; CI: confidence interval

### Subgroup analysis of the primary and secondary outcomes

After covariate adjustment, AHRQ-PPC was associated with upper endoscopy [5.9 vs. 1.0%, OR_adj_: 3.76, 95%CI (1.85, 7.66), p<0.001]. When compared to gastroenterologist-guided conscious sedation, anesthesia care team presence was protective against AHRQ-PPC [3.7 vs. 8.4%, OR_adj_: 0.032, 95%CI (0.01, 0.22), p<0.001] and AHRQ-ASP [1.0 vs. 3.37%, OR_adj_: 0.002, 95%CI (0.00, 0.55), p<0.001] but not for AHRQ-PNA [2.0 vs. 4.0% OR_adj_: 0.238, 95%CI (0.03, 1.96), p = 0.18].

## Discussion

We report risk estimates for 30-day PPC in a diverse patient population undergoing GIEP, utilizing the AHRQ-PPC composite. Risk estimates utilizing this broad composite of 30-day PPC have not been previously published and this study provides insight into their expected incidence. Overall, we observed a 30-day incidence of AHRQ-PPC of 4.3%, AHRQ-PNA (2.3%), AHRQ-RF (1.7%) and AHRQ-ASP (1.37%). When categorized by location, outpatient GIEP demonstrated lower incidence of both the primary (AHRQ-PPC), and secondary outcomes (AHRQ-RF, AHRQ-PNA, AHRQ-ASP), as compared to inpatient GIEP. Furthermore, the involvement of an anesthesia care team was protective towards the occurrence of 30-day PPC.

Broad-based PPC composites may not clearly illustrate the direct impact of the endoscopy procedure itself, due to inevitable/inseparable confounders associated with inpatient admission. Outpatient procedures are more commonly performed on an elective as opposed to urgent basis and patients tend to be relatively healthier. Due to these factors, it is likely that outpatient 30-day PPC incidence is a more precise risk estimate attributable to GIEP. Furthermore, 26.6% of AHRQ-PPC were recorded after discharge from the inpatient admission or outpatient procedure. This is consistent with the findings of Aasen and colleagues regarding the high incidence of post-discharge infectious complications, thus identifying 30-day PPC, vs. admission related, findings may provide an improvement in risk identification and opportunity for mitigation [6].

In the AHRQ-PNA sub-composite, we observed a 3.2% (inpatient) and 0.7% (outpatient) 30-day incidence of infectious pneumonia, after GIEP. When compared to general estimates in surgical populations, this is lower than the reported incidence of inpatient postoperative pneumonia published in the Nationwide Inpatient Sample (NIS) and National Surgical Quality Improvement Program (NSQIP) [3], although comparisons are limited by different time frames. Similarly, we observed a 30-day AHRQ-ASP incidence of 0.4% after outpatient GIEP. Although our database lacks the ability to ascertain cause and effect in regard to pulmonary aspiration, evidence of silent, unrecognized pulmonary aspiration during endoscopic procedures has been previously documented at 3.94% as incidental findings on F-FDG PET scanning for routine cancer screening [11]. In contrast, a prospective pilot study using ingested nonabsorbable technetium-99m did not find meaningful pulmonary aspiration in upper GIEP [12].

We observed that 30-day AHRQ-PPC was strongly associated with pre-procedural Elixhauser comorbidities of electrolyte disorder, alcohol use disorder, CHF and pulmonary circulatory disorders. Though composite outcomes are less precise for the purposes of risk prediction, these findings are consistent with published data suggesting increased risks for PPC in CHF, alcohol use disorders, and pulmonary hypertension [13–17]. Acute alcohol consumption and alcohol related liver disease have been shown to predispose to tracheobronchial aspiration and it is probable that patients with these conditions have higher risk for 30-day PPC [18]. Similarly, patients with CHF and pulmonary circulatory disorders have higher risk for postoperative respiratory failure related to periprocedural volume shifts among other factors [19]. Lastly, renal dysfunction and electrolyte disorders surrounding perioperative care are well established. Although pre-operative renal impairment has not generally been associated with 30-day mortality, 30% of surgical inpatients develop complications related to fluid and electrolyte therapy. Regarding endoscopic procedures, many involve using fluid irrigation to improve visualization with potential for significant systemic absorption of fluid. Sodium and water retention remains a common source of perioperative morbidity related to increased incidence of pulmonary edema, concomitant respiratory insufficiency and cardiac arrhythmias [20]. Similarly, both acquired and pre-admission serum sodium abnormalities have been associated with increased hospital mortality [21]. Our findings may suggest that enhancing pre-procedural optimization of electrolyte disorders, more protocolized vigilance for early signs of impaired respiratory mechanics and closer monitoring for post-procedural pulmonary congestion may improve 30-day PPCs after GIEP.

Upon exploratory analysis, we identified that upper GIEP were significantly higher risk for 30-day AHRQ-PPC with a 5.9% vs. 1% incidence for lower GIEP. It seems evident that airway manipulation during upper endoscopic instrumentation is more likely to generate PPC than lower GI tract endoscopic instrumentation. However, we believe that this observation permits system processes to focus limited resources for protocolized PPC risk mitigation on this procedure type. After adjustment, anesthesia care team presence appeared to be protective, nearly halving 30-day PPC risk (3.7% vs. 8.4%) and reducing sub-composite AHRQ-ASP events (1.0% vs. 3.37%). Pneumonia risk was reduced (2 vs. 4%) but was not statistically significant. Selection criteria for anesthesia care team presence for non-operating room procedures remain an evolving science and have remained focused on specific procedures [22, 23]. To our knowledge, no studies have examined the direct effect of anesthesia care team presence on 30-day PPC. However, there is indirect evidence that may support our observation. A meta-analysis by Leung and colleagues, comparing general endotracheal anesthesia vs. sedation for endoscopic submucosal dissection, found superior outcomes with anesthesiologist directed care, including reduced rates of gastrointestinal perforation and post-procedural aspiration pneumonia [24]. One study comparing general endotracheal anesthesia to monitored anesthesia care (MAC) in patients presenting for endoscopic retrospective cholangiopancreatography (ERCP) observed higher sedation-related adverse events in the MAC group, predominantly driven by frequent need for airway maneuvers, furthermore, this resulted in increased procedural interruptions for definitive management [25]. Lamireau and colleagues observed higher rates of significant hypoxia in pediatric patients undergoing esophagogastroduodenoscopy under sedation when compared to general anesthesia [26]. One study suggested equivocal differences when comparing moderate sedation vs. monitored anesthesia care with endpoints focused primarily on post-ERCP complications [27]. We observed that anesthesia care team presence reduces overall 30-day PPC, with a relative risk reduction of 55% (RR: 0.4474, Numbers Needed to Treat (NNT): 21.658), potentially providing downstream cost and safety benefits to GIEP. Given the costly nature of PPC to healthcare systems, these findings should be further explored. It is important to note that this observation is limited by our lack of database granularity regarding the physical environment for nursing/gastroenterology performed procedures and the selected anesthesia management, particularly endotracheal intubation.

Limitations of this study are consistent with those found in retrospective cohort studies, namely, potential coding errors, observer bias, selection bias and convenience sampling. It is important to note that unmeasured confounding factors may be present that could modify the direct contribution of PPC to patient morbidity and mortality after GIEP. This is particularly salient with inpatient GIEP, as it difficult to identify the direct contribution of GIEP to AHRQ-PPC in this population. Furthermore, it is important to note that direct causality is difficult to identify when examining 30-day outcomes, particularly given that readmission after GIEP may not be directly associated with procedural complications. To reduce these biases, we curated a diverse population where data extraction was performed by ICD-10 and CPT code by an independent data analytics team. These interventions may partially mitigate these risks. Furthermore, we balanced clinically relevant baseline covariates using PSM for preoperative risk predictors and adjusted for clinically relevant covariates to enhance the precision of our outcome observations. Lastly, it is important to stress that sub-composite findings are limited by the presence of overlapping codes, for example, a patient with ventilator-associated pneumonia may have ICD-10 and CPT codes for both respiratory failure (AHRQ-RF) and infectious pneumonia (AHRQ-PNA).

## Conclusions

In conclusion, these findings report general risk estimates of 30-day PPC after GIEP across a broad range of inpatient and outpatient settings in a large healthcare system. We observed higher 30-day PPC in patients with comorbidities of electrolyte disorders, alcohol abuse, CHF and pulmonary circulatory disorders. We confirmed that upper and inpatient GIEP comprise the majority of 30-day PPC risk but direct causation to GIEP is difficult. Outpatient GIEP had a 1.2% incidence of 30-day AHRQ-PPC, an incidence of 0.7% AHRQ-PNA and 0.4% incidence of AHRQ-ASP. In this study cohort, 26.6% of 30-day PPC occurred after discharge, suggesting that a large portion of PPC are not identified during hospital stay. Lastly, the presence of anesthesia care teams may reduce the risk for 30-day PPC and tracheobronchial aspiration events, potentially reducing downstream complications and their associated healthcare costs. Future studies should seek to further evaluate specific medical conditions or pre-procedural interventions that may contribute or mitigate 30-day PPC risk, respectively, especially across multiple centers.

## Supporting information

S1 Table International classification of disease (Version 10) codes and sub-composite variables utilized for the primary and secondary outcomes. (DOCX)
