# Supplementary material for: Estimates of 30-day postoperative pulmonary complications after gastrointestinal endoscopic procedures: A retrospective cohort analysis of a health system population
Source: PLoS One. 2024 Feb 23;19(2):e0299137. doi: 10.1371/journal.pone.0299137 (PMC10889900; doi:10.1371/journal.pone.0299137)
Supplement: S1 Table — (DOCX) [file pone.0299137.s001.docx]

| **Supplementary Table 1.** | | |
| --- | --- | --- |
| **30-day AHRQ-PPC** | **ICD-10** | **Sub-Composite Inclusion** |
| Transfusion-related acute lung injury | J95.84 | AHRQ-RF |
| Respiratory complications not elsewhere classified | J95.85 | AHRQ-RF |
| Ventilator associated pneumonia | J95.86 | AHRQ-PNA |
| Post-procedural aspiration pneumonia | J95.87 | AHRQ-ASP |
| Other complication of ventilator | J95.88 | AHRQ-RF |
| Other intraoperative complications of respiratory system, not elsewhere classified | J95.89 | AHRQ-RF |
| Other postprocedural complications and disorders of respiratory system, not elsewhere classified | J95.90 | AHRQ-RF |
| Acute respiratory failure, unspecified whether with hypoxia or hypercapnia | J95.91 | AHRQ-RF |
| Respiratory failure, unspecified, unspecified whether with hypoxia or hypercapnia | J95.92 | AHRQ-RF |
| Respiratory failure, unspecified with hypoxia | J95.93 | AHRQ-RF |
| Respiratory failure, unspecified with hypercapnia | J95.94 | AHRQ-RF |
| Acute and chronic respiratory failure, unspecified whether with hypoxia or hypercapnia | J95.95 | AHRQ-RF |
| Acute and chronic respiratory failure with hypoxia | J95.96 | AHRQ-RF |
| Acute and chronic respiratory failure with hypercapnia | J95.97 | AHRQ-RF |
| Respiratory disorders in diseases classified elsewhere | J95.98 | AHRQ-RF |
| Pulmonary insufficiency following trauma and surgery | J95.99 | AHRQ-RF |
| Acute postprocedural respiratory failure | J95.100 | AHRQ-RF |
| Acute respiratory failure with hypoxia | J95.101 | AHRQ-RF |
| Acute respiratory failure with hypercapnia | J95.102 | AHRQ-RF |
| Acute pulmonary insufficiency following thoracic surgery | J95.103 | AHRQ-RF |
| Acute pulmonary insufficiency following nonthoracic surgery | J95.104 | AHRQ-RF |
| Chronic pulmonary insufficiency following surgery | J95.105 | AHRQ-RF |
| Acute and chronic postprocedural respiratory failure | J95.106 | AHRQ-RF |
| Acute respiratory distress syndrome | J95.107 | AHRQ-RF |
| Chronic respiratory failure, unspecified whether with hypoxia or hypercapnia | J95.108 | AHRQ-RF |
| Chronic respiratory failure with hypoxia | J95.109 | AHRQ-RF |
| Chronic respiratory failure with hypercapnia | J95.110 | AHRQ-RF |
| Respiratory arrest | J95.111 | AHRQ-RF |
| Pneumonia due to streptococcus pneumonia | J95.112 | AHRQ-PNA |
| Other bacterial pneumonia | J95.113 | AHRQ-PNA |
| Pneumonia due to Pseudomonas | J95.114 | AHRQ-PNA |
| Pneumonia due to streptococcus | J95.115 | AHRQ-PNA |
| Pneumonia due to staphylococcus | J95.116 | AHRQ-PNA |
| Pneumonia due to Methicillin susceptible Staphylococcus | J95.117 | AHRQ-PNA |
| Pneumonia due to Methicillin susceptible Staphylococcus aureus | J95.118 | AHRQ-PNA |
| Pneumonia due to Escherichia coli | J95.119 | AHRQ-PNA |
| Pneumonia due to other Gram-negative bacteria | J95.120 | AHRQ-PNA |
| Pneumonia due to other specified bacteria | J95.121 | AHRQ-PNA |
| Unspecified bacterial pneumonia | J95.122 | AHRQ-PNA |
| Pneumonia due to other specified infectious organisms | J95.123 | AHRQ-PNA |
| Invasive pulmonary aspergillus | J95.124 | AHRQ-PNA |
| Bronchopneumonia, unspecified organism | J95.125 | AHRQ-PNA |
| Pneumonia, unspecified organism | J95.126 | AHRQ-PNA |
| Pneumonia due to Klebsiella pneumonia | J95.127 | AHRQ-PNA |
| Pneumonia due to other streptococci | J95.128 | AHRQ-PNA |
| Pneumonitis due to solids and liquids | J95.129 | AHRQ-ASP |
| Hypostatic pneumonia, unspecified organism | J95.130 | AHRQ-PNA |
| Other ill-defined and unknown causes of morbidity and mortality | J95.131 |  |
| Respiratory conditions due to chemical fumes and vapors | J95.132 |  |
| Pneumonitis due to solids and liquids | J95.133 | AHRQ-ASP |
| Pneumonitis due to inhalation of food and vomit | J95.134 | AHRQ-ASP |
| Postprocedural pneumothorax | J95.135 |  |
| Septic pulmonary embolism without acute cor pulmonale | J95.136 | AHRQ-PE |
| Other pulmonary embolism without acute cor pulmonale | J95.137 | AHRQ-PE |
| Air embolism following infusion, transfusion and therapeutic injection, initial encounter | J95.138 |  |
| Complication of other artery following a procedure, not elsewhere classified, initial encounter | J95.139 |  |
| Complication of vein following a procedure, not elsewhere classified, initial encounter | J95.140 |  |
| Embolism due to cardiac prosthetic devices, implants and grafts, initial encounter | J95.141 | AHRQ-PE |
| Embolism due to cardiac prosthetic devices, implants and grafts, initial encounter | J95.142 | AHRQ-PE |
| Embolism due to vascular prosthetic devices, implants and grafts, initial encounter | J95.143 | AHRQ-PE |
| Embolism due to vascular prosthetic devices, implants and grafts, initial encounter | J95.144 | AHRQ-PE |
| Abbreviations : AHRQ: Agency for Healthcare Research & Quality; AHRQ-PPC: AHRQ total postoperative pulmonary complication composite; AHRQ-RF: AHRQ respiratory failure sub-composite; AHRQ-ASP: AHRQ aspiration pneumonitis/pneumonia sub-composite; AHRQ-PNA: AHRQ pneumonia sub-composite. | | |
